# Maternal Antiretroviral Use and the Risk of Prematurity and Low Birth Weight in Perinatally HIV-Exposed Children—7 Years’ Experience in Two Romanian Centers

**DOI:** 10.3390/medicina62010093

**Published:** 2026-01-01

**Authors:** Ana Maria Tudor, Simona Claudia Cambrea, Laurențiu Mihăiță Stratan, Constanța Angelica Vișan, Cătălin Tilișcan, Victoria Aramă, Simona Maria Ruță

**Affiliations:** 1 Faculty of Dentistry, “Carol Davila” University of Medicine and Pharmacy, 050474 Bucharest, Romania; angelica.visan@umfcd.ro; 2 “Prof. Dr. Matei Balş” National Institute of Infectious Diseases, 021105 Bucharest, Romania; catalin.tiliscan@umfcd.ro (C.T.); victoria.arama@umfcd.ro (V.A.); 3 Faculty of Medicine, “Ovidius” University from Constanta, 900470 Constanta, Romania; 4 Clinical Hospital of Infectious Diseases Constanta, 900709 Constanta, Romania; 5 Faculty of Medicine, “Carol Davila” University of Medicine and Pharmacy, 050474 Bucharest, Romania; simona.ruta@umfcd.ro; 6 Department of Emerging Viral Diseases, Stefan S. Nicolau Institute of Virology, Romanian Academy, 030304 Bucharest, Romania

**Keywords:** HIV, antiretroviral drugs, prematurity, low birth weight

## Abstract

*Background and Objectives:* Antiretroviral therapy used during pregnancy in HIV infected women effectively reduces vertical transmission, though concerns about potential adverse newborn outcomes persists. This study focused on prematurity and low birth weight in antiretroviral HIV-exposed children in two major Romanian centers, Bucharest and Constanța, in the context of free access to antiretroviral treatment for pregnant women in Romania since 2001. *Materials and Methods:* A retrospective observational study was performed including couples of HIV-infected women and their live singleton newborns from 2006 and 2012. Preterm delivery was defined as birth before week 37 and low birth weight was defined as birth weight less than 2500 g in full-term babies. *Results:* A total number of 352 children and 313 women were enrolled. Mean maternal age at delivery was 23.1 years. Mean newborn birth weight was 2726 g. In the children group, 191 (54.2%) were boys, and the rate of HIV transmission was 13.9%. The prematurity rate was 21.5% and low birth weight rate was 25.56%. Preterm birth was associated with high HIV RNA in the third trimester, HIV-positive final status in infants, and vaginal delivery. Low birth weight was associated with lack of antiretroviral treatment during pregnancy and HIV-positive status in infants. No association was found between prematurity and low birth weight in full-term newborns and exposure to any antiretroviral class, any specific antiviral drug, or with any number of maternal regimens, duration of antiretroviral treatment prior to conception, or maternal exposure during puberty. *Conclusions:* In our study, preterm birth was significantly associated with HIV vertical transmission in newborns and with exposure to high maternal viral replication during the last trimester of pregnancy. Low birth weight in full-term babies was significantly associated with lack of antiretroviral exposure in utero in our analysis.

## 1. Introduction

There is much scientific data regarding the positive effect of combined antiretroviral treatment (cART) on the evolution of HIV infection, with consistent increases observed in the survival time and quality of life of HIV-infected individuals [1,2]. Antiretroviral medicines reduce the risk of viral transmission, including between a mother and her child during pregnancy, labor and breastfeeding. Nevertheless, concerns exist regarding the effects of antiretroviral drugs on the offspring of treated HIV-infected women, more so if the maternal antiretroviral history includes a high number of regimens and exposure during puberty [3,4,5].

The Romanian National Program for HIV Treatment and Prevention started in 2001 and provides free-of-charge HIV testing for pregnant women, efficient antiretroviral regimen for all infected individuals, and antiretroviral prophylaxis to all newborns from HIV-infected women. Epidemiologic data from Romania published by the department for monitoring and evaluation of HIV/AIDS infection in Romania show a constant downward trend of vertical transmission rate from 11% in 2010 to 4% in 2015 to 3% in 2024 [6].

The efficacy of antiretroviral treatment in preventing HIV vertical transmission is reported by numerous clinical studies. A recent systematic review and meta-regression analysis published in September 2025 in Lancet HIV showed a dramatic decrease in HIV vertical transmission since 2013 when the World Health Organization (WHO) issued a recommendation for combined antiretroviral treatment (cART) immediately, at any gestational age during pregnancy, with continuation for life [7].

A systematic review published in 2025 estimated the risk of perinatal vertical transmission with no prophylactic measures at 33.4%, compared to 5.6% in women receiving cART in the last month of pregnancy and 0.3% in those started on cART before pregnancy. At the same time, the efficacy of HIV vertical transmission prevention was not significantly different in regard to the various antiretroviral regimens analyzed by the review [8].

While the crucial importance of antiretroviral drugs in mother to child HIV transmission is well established, the negative impact on pregnancy and offspring is still debated. The panel of experts reviewing the USA-approved guidelines for HIV infections updated the recommendations for HIV treatment during pregnancy in June 2025, and they stated that despite the fact that many studies have assessed the association of antiretroviral drugs with undesirable birth outcomes, like premature delivery, low newborn weight at birth, and stillbirth, the results are conflicting, so there is still need for more information on this matter [9].

Moreover, several large studies have shown contradictory results on the potential teratogenic effects of antiretrovirals (ARVs) used for prevention of HIV mother-to-child transmission (PMCT). A large French cohort study including more than 13,000 children exposed to ARVs, followed up since 1994, showed an association between the use of zidovudine during the first trimester of pregnancy and the incidence of congenital heart defects, as well as between the use of efavirenz and the appearance of neural tube defects [10].

Other studies, while not identifying any major teratogenic risks associated with the use of ARVs, before and during pregnancy, signaled a higher risk of prematurity in newborns exposed in utero to protease inhibitors (PIs) [11,12].

The rate of prematurity was also found to be higher in HIV-infected pregnant women versus an uninfected group by researchers from Barcelona, although they did not observe a higher rate of complications associated with mitochondrial toxicity, suggesting that HIV by itself is a risk factor for preterm delivery [13].

An Italian cohort study from 2014, the ICONA Study, found the rates of premature birth to be 11.9% in ARV-naïve patients and 22.6% in ARV-treated patients, but no association with duration of antiretroviral treatment or with protease inhibitors was observed [14].

Nevertheless, in a German cohort of 183 mother–child pairs, the risk of prematurity was 3.4 for PI-based cART exposure during pregnancy compared with ARV monotherapy, and congenital anomalies were found in 3.3% of births [15].

The epidemiology of HIV infection in Romania is characterized by the presence of a high number of perinatally infected patients with HIV-1 subtype F [16,17] who become sexually active and have their own children. The rate of vertical transmission in Romania has remained low, less than 5%, and stable over the last 5 years, mainly due to a National Program for Prevention of Mother to Child HIV Transmission, in place from 2002, which consists of three ARVs, usually a backbone combination of two nucleoside reverse transcriptase inhibitors (NRTIs) with either a boosted protease inhibitor (PI/r), non-nucleoside reverse transcriptase inhibitor (NNRTI), or integrase inhibitor (INSTI) administered to the pregnant women from diagnosis, with cesarean section delivery, avoidance of breastfeeding, and antiretroviral prophylaxis of newborns for 6 weeks after birth [18].

The impact of antiretroviral drugs on the offspring of a Romanian cohort of multi-ARV-treated patients was assessed by several studies recently published. One study was conducted in Galati County, including 114 in utero antiretroviral-exposed newborns, with premature birth in 28.07%, 8.7% with significant birth defects, and three deaths, higher compared to national or European registries, but similar with reported data in HIV-infected pregnant women from other Romanian Centers in Bucharest and Constanta, 19% and 31%, respectively [19,20].

In another Romanian study including 408 HIV-exposed newborns in two centers in Craiova and Constanta, high rates of fetal growth restrictions were observed, similar in the studied centers at around 50%, higher in HIV-positive newborns at 55–58% and lower in HIV-negative ones at 45–50% [21].

The study presented in this paper aimed to assess the associations between two aspects of adverse birth outcomes, preterm delivery and low birth weight, in full-term newborns, as well as maternal HIV infection markers (HIV RNA and CD4 blood levels at delivery) and antiretroviral in utero exposure.

A secondary objective was to assess the associations between preterm birth and low birth weight in full-term babies and maternal antiretroviral history, specifically the duration of cART, number of regimens, types of regimens, and exposure during maternal puberty. This study will add information to a field with limited and controversial data in a country with free access to antiretroviral treatment and free HIV testing for pregnant women. Moreover, this study assessed the risk of prematurity and low birth weight and maternal antiretroviral history, including during childhood and puberty, aspects not studied yet to the best of our knowledge.

## 2. Materials and Methods

A retrospective observational study was performed on HIV-infected women and their offspring monitored in two of the largest regional centers for HIV infection in Romania, National Institute for Infectious Diseases “Prof. dr. Matei Bals” Bucharest and Clinical Hospital of Infectious Diseases Constanta.

The Ethical Committee approved the study. Informed consent was signed by all patients or their legal guardians. (Ethical Council approval 14 November 2025 C12987, Bioethics Committee approval 19 November 2025 C13162.)

Patients’ assessment conformed to the Romanian National HIV guidelines at the moment of diagnosis [18]. HIV-infected women were diagnosed based on ELISA testing, confirmed with Western blot tests, plus a detectable level of HIV RNA prior to cART. Assessment of treatment during pregnancy for women and medical care for children was conducted by infectious disease specialists as individual case managers [18].

The final HIV status in HIV-exposed children was recorded as HIV infection at first detectable HIV RNA level (viral load or viremia) during a follow-up period of 18 months. Uninfected status in HIV-exposed children was considered if, at 18 months of age, the viral load was undetectable without antiretroviral treatment and HIV antibodies tested negative using ELISA and Western blot tests. Undetermined status was considered in HIV-exposed children with no information about viral load at the end of follow-up period, but with undetectable HIV RNA without antiretroviral treatment at the last available evaluation. Patients with no information about viral load, ELISA, or Western blot results at the end of the study were registered as lost to follow-up. All offspring of one mother were included if they were born within the study period.

Information about the patients was retrieved from medical files, and reflected the common medical HIV care provided in Romania. Data collected included the gestational and maternal age at birth, treatment during pregnancy, and duration of cART before pregnancy, as well as newborn gender, birth weight, and antiretroviral prophylaxis.

All infants were evaluated at birth and at 3, 6, 12, and 18 months to determine their HIV status.

HIV viral load was determined using the commercial kit COBAS TaqMan HIV-1 Test Version 2.0, Roche Molecular Systems, Branchburg, NJ, USA; lower detection limit: 20 HIV RNA (viremia) copies/mL. The viremia levels were stratified as virologic success for less than 50 copies, 50–200 copies/mL, and higher than 200 copies/mL. Lymphocytes’ CD4 levels were stratified as less than 200 cells/mm^3^, considered as severe immunosuppression, 200–500 cells/mm^3^, indicating moderate immunosuppression, and higher than 500 cells/mm^3^, reflecting no immunosuppression [7].

Gestational age (GA) was determined based on date of the last period and/or ultrasound measurements of the fetus. If these data were not available, the gestational age was determined using the Ballard scoring system [22,23]. A normal gestational period of 37 to 40 weeks was considered as full-term birth (FB), and normal birth weight (NBW) was greater than 2500 g. Premature birth (PB) was noted if delivery occurred before the end of 37 weeks of gestation or Ballard score was less than 35, while low birth weight (LBW) was noted if the newborn weighed less than 2500 g.

Maternal comorbidities assessed in this study were chronic HBV or HCV infection, illegal drug use, and syphilis diagnosis during pregnancy. Type of delivery was stratified as vaginal and planned or necessary cesarean section.

The duration of cART in the studied women prior to pregnancy was stratified as follows: greater than 120 months, 60 to 119 months, 12 to 59 months, and less than 12 months. Stratification took into account the mean maternal age from our study and mean age of puberty. Antiretroviral exposure during puberty was assessed indirectly, taking into account the mean maternal age at birth and duration of cART prior to conception longer than 10 years (120 months) [24].

The regimens used during pregnancy were stratified as NNRTI or PI-based combination.

Statistical analysis was performed using Epi Info 7 for Microsoft Windows Open Epi, Version 3, open-source calculator from the Centers for Disease Control and Prevention (CDC) website at https://www.openepi.com/Menu/OE_Menu.htm last (accessed at 25 November 2025). The Pearson’s chi square test was used to assess the differences between sets of categorical variables and the Mann–Whitney test was used for non-parametric variables. Statistical significance (*p*) was computed using the Fisher test for categorical variables, Student *t* test, and ANOVA for means, and statistical significance was considered at less than 0.05.

## 3. Results

### 3.1. Study Population

#### 3.1.1. Description of Studied Children

The number of children included in the study was 352. During the study period, 31 women out of 313 had two pregnancies and 6 out of 313 had three pregnancies. No death among the studied women or newborns was recorded.

The characteristics of the studied newborns showed balanced distribution by gender; 191 (54.2%) were boys.

The low birth rate in all studied newborns was 25.56% (90 cases out of 352), higher in preterm babies 75% (57 out of 76 cases) compared to full-term babies 12.26% (33 out of 269 cases), as expected.

**Figure 1:**
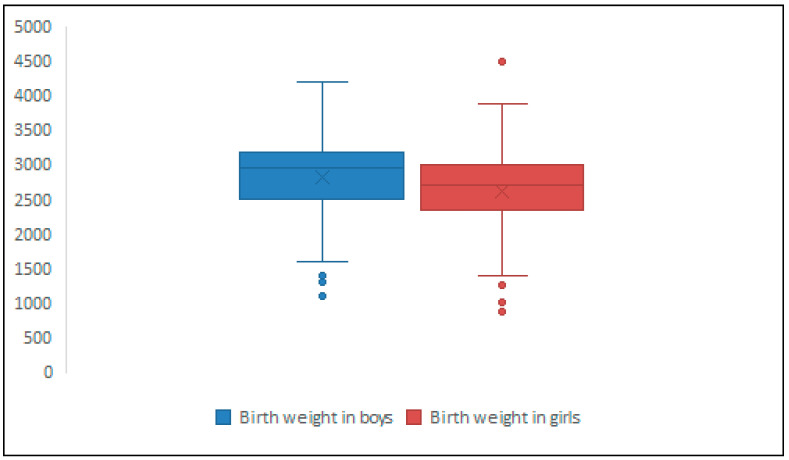
Distribution of birth weight by gender in studied population. Legend> “X” marks the mean value of the dataset and circles represents outliers, data points below and above the whiskers (more than 1.5 times the interquartile range from the quartiles).

The mean birth weight was 2726 g, with a standard deviation (SD) of ±555 g and 95% confidence interval (95%CI) of 2667.8–2784.2. The mean birth weight distribution by gender was 2615 g (SD ± 525, 95%CI: 2533.3–2696.7) in girls and 2817 g (SD ± 562, 95%CI 2736.8–2897.2) in boys, and the difference was statistically significant (*p* < 0.001, t statistics 3.46–4.48, degree of freedom 346–350, mean difference 202, 95%CI 87.23–316.76 for equal variance), showing that girls are more likely to have lower birth weight, consistent with normal biologic distribution by gender (Figure 1).

The highest birth weight observed was 4500 g in a HIV-positive girl, delivered by C section at 40 weeks of gestation by a HIV-positive mother diagnosed during delivery, with no comorbidities and no antiretroviral treatment during pregnancy. The lowest recorded birth weight was 870 g in a HIV-negative girl delivered at 28 weeks of gestation by a HIV-positive mother, diagnosed 5 years before conception, with a viral load of 4 log in the last trimester, who was an iv drug user and HCV-coinfected, nonadherent to antiretrovirals.

The rate of HIV transmission among studied babies was 13.9%, namely 49 out of 352 enrolled children, but 39 (11%) HIV-exposed infants were lost to follow-up. Although these infants were not evaluated at 18 months of age, they had an undetectable viral load at the last available determination. After performing a sensitivity test for missing data under the assumption that data were not missing at random (MNAR), the transmission rate yielded from 14.2% to 16.4%. The lowest estimate was reported due to 95% confidence intervals overlapping, and shift estimates (SEs) were less than 2% for the following three scenarios: missing data at random (MAR delta adjustment, δ = 0, SE = 1.92%, 95%CI = 11.6–19%), low MNAR (scale factor conservative, c = 0.8, δ = −0.05, SE = 1.95%, 95%CI 10.4–18%), and high MNAR (c = 1.2, SE = 1.90%, 95%CI = 12.8–20%).

Preterm delivery was recorded in 76 (21.59%) newborns and low birth weight in 91 (25.85%) newborns. Missing data on gestational age was noted in 7 cases (1.98%) and on birth weight in 14 babies (3.97%).

#### 3.1.2. Description of Maternal Characteristics

The mean maternal age at delivery was 23.1 years (SD ± 4.4 years, 95%CI 22.6–23.5). Most of the women enrolled in the study were diagnosed before conception, 219 out 352 (62.21%), and 185 out of 352 (52.55%) were on antiretroviral treatment before pregnancy.

The viral load in the last trimester of pregnancy was below 50 copies/mL in 81 out of 352 (23.01%) women, 51 to 200 copies/mL was found in 11 (3.12%) women, 29 (8.23%) had an HIV RNA level between 201 and 1000 copies, 129 (36.64%) had over 1000 copies/mL, and in 102 women (28.97%), no data about viremia was recorded.

Distribution of CD4 cells levels in the last trimester of gestation in the studied women showed CD4 levels below 200 cell/mm^3^ in 46 out of 352 (13.06%), 123 (34.94%) had a CD4 count of 200–500 cell/mm^3^, 118 (33.52%) cases noted over 500 cell/mm^3^, and 65 (18.46%) cases had no data regarding CD4 levels in the last trimester of pregnancy.

**Figure 2:**
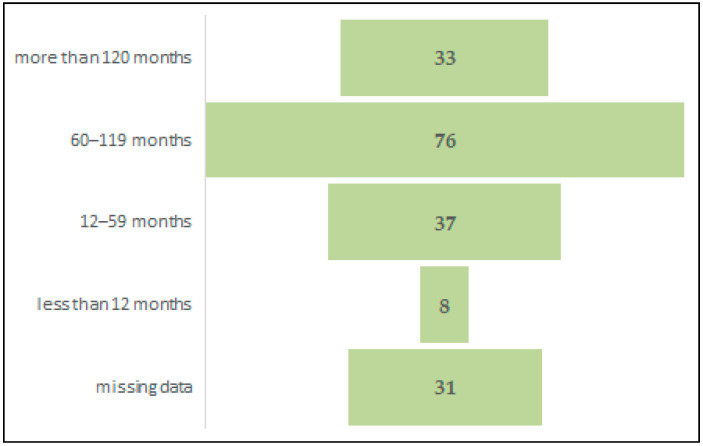
Distribution of maternal cases by cART duration before pregnancy.

Distribution of maternal cases by cART duration before pregnancy showed that most of the studied women, 76 out of 185 (41.08%), initiated cART between 60 and 119 months before conception, 33 out of 185 (17.83%) had therapy for more than 120 months before conception, 37 (20%) were treated between 12 and 59 months before pregnancy, and 8 (4.32%) less than one year before conception. In 31 (16.75%) maternal cases, there were no recorded data about timing of cART initiation. (Figure 2). The longest duration of antiretroviral therapy prior to conception was 176 months (almost 15 years), and the shortest was two months.

The mean number of regimens in pretreated women was three (SD = 1.376, 95%CI = 2.807–3.192, *p* = 0.00614), while in the overall studied maternal population, the mean was 1 (SD = 1.62, 95%CI = 0.82–1.162 *p* = 0.0062). The highest number of antiretroviral regimens recorded in the studied women was seven.

In the studied cases, 58 (16.47%) pregnant women started therapy after conception. The total number of newborns exposed to antiretrovirals in utero was 239 out of 352 (67.89%), with 183 out of 239 (76.56%) being exposed from the first trimester. Distribution of the antiretroviral regimen type used during pregnancy in the studied patients revealed a predominance of protease-inhibitor-based combinations, in 221 (92.46%) out of 239 cases, while only 9 out 239 (3.76%) underwent non-nucleoside reverse transcriptase inhibitor-based regimens and 9 out of 239 (3.76%) underwent a combination of PI and NNRTI. In three cases, there were two PIs used due to switching therapy during pregnancy, from saquinavir to lopinavir (two cases) and darunavir (one case). Finally, there were 224 exposures to PIs and 18 to NNRTIs.

Analyzing the type of molecules used, in the PI class, the most used was boosted lopinavir in 165 out of 224 (73.66%), 12 (5.35%) used nelfinavir, 38 (16.96%) cases used boosted saquinavir, 7 (3.12%) cases used boosted darunavir, and 2 (0.89%) cases used boosted atazanavir.

The studied newborns were exposed to the following NNRTI molecules: efavirenz in 8 out of 18 (44.44%) cases, and nevirapine and etravirine in 5 (27.78%) cases each.

The backbone regimens were represented by combination of nucleotide/nucleoside reverse transcriptase inhibitors, namely zidovudine plus lamivudine in 189 out of 239 (79.07%) cases, abacavir plus lamivudine in 25 (10.46%) cases, tenofovir plus lamivudine in 5 cases (2.09%), abacavir plus didanosine in 8 (3.34%) cases, and other combinations in 12 (5.02%) cases.

#### 3.1.3. Prematurity Assessment

Univariate analysis was performed to assess the association of prematurity with newborn characteristics, gender, and final HIV status and the following maternal characteristics: mean age, comorbidities, type of delivery, virologic and immunologic markers, namely HIV RNA and CD4 lymphocytes levels during the last trimester of pregnancy, antiretroviral exposure during pregnancy, duration of maternal antiretroviral treatment before conception, type of antiretrovirals used, and timing of cART during gestation. In seven children, information about gestational age was missing. The data is presented in Table 1.

Preterm birth distribution was similar between genders (*X*^2^ (2, 352) = 2.081, *p* = 0.35) (Table 1). The final HIV status of the infants was correlated with prematurity, *X*^2^ (4, *N* = 352) = 18.07, *p* = 0.001), meaning that an HIV-infected newborn was more likely to be delivered prematurely in the studied patients.

In this study, premature newborns were more likely to be associated with vaginal delivery. Otherwise, full-term birth was more likely to be found in cases of planned C section and vaginal delivery (*X*^2^ (6, *N* = 352) = 127.3, *p* < 0.0000001).

Univariate analysis of prematurity’s association with maternal characteristics revealed that premature delivery was more likely to be found in pregnant women with HIV RNA over 1000 copies/mL in the third trimester of gestation (*X*^2^ (8, *N* = 352) = 22.13, *p* = 0.004). Timing of HIV maternal antiretroviral therapy before, during pregnancy, and at or after birth showed an uneven distribution in the studied groups, driven by missing data, so the results did not permit a valid interpretation (*X*^2^ (6, *N* = 352) = 17.76, *p* = 0.007).

In the studied patients, there was no association between prematurity rate and timing of maternal HIV diagnosis, before or after conception (*X*^2^ (4, *N* = 352) = 5.25, *p* = 0.26), maternal immunity status stratified by CD4 count levels (*X*^2^ (6, *N* = 352) = 11.196, *p* = 0.08), antiretroviral exposure during first trimester (*X*^2^ (6, *N* = 352) = 7.16, *p* = 0.30), number of antiretroviral regimens in treated women (*X*^2^ (10, *N* = 352) = 9.33, *p* = 0.50), or maternal comorbidities, namely chronic HBV infection (*X*^2^ (2, *N* = 352) = 0.51, *p* = 0.77), chronic HCV infection (*X*^2^ (2, *N* = 352) = 2.55, *p* = 0.27), illegal drug use (*X*^2^ (2, *N* = 352) = 1.02, *p* = 0.60), and syphilis diagnosis during pregnancy (*X*^2^ (2, *N* = 352) = 4.25, *p* = 0.11). Obstetrical complications were noted in two cases of premature delivery and none in the full-term delivery group, revealing a statistically significant difference explained by a plausible medical association between obstetrical complications and premature birth.

Regarding exposure to antiretrovirals during pregnancy, the statistical analysis showed a significant difference in distribution among studied newborns with no data about gestational age, precluding a meaningful interpretation of the results (*X*^2^ (4, *N* = 352) = 9.77, *p* = 0.04) (Table 1).

In women treated before conception (185 cases), there was no association between premature delivery and duration of antiretroviral therapy before pregnancy, stratified as follows: more than 120 months, 60 to 199 months, 12 to 59 months, and less than 12 months (*X*^2^ (8, *N* = 185) = 7.91, *p* = 0.44).

The mean duration of maternal treatment before pregnancy in those treated for more than 120 months was 145 ± 15.6 (95%CI: 138.7–151.2) months in full-term babies and 137.9 ± 11.8 months (95%CI: 129.1–146.6) in preterm babies, and the difference was not statistically significant (equal variance *t* = 1.17, *p* = 0.24, 95%CI −5.24–19.44 and unequal variance *t* = 1.35, *p* = 0.19, 95%CI −4.02–18.22). In a univariate analysis, antiretroviral exposure (stratified as more and less than 120 months) had no impact on prematurity rate (*p* = 0.52, OR 2.6638, Risk Ration 1.08, Risk difference 1.77).

The antiretrovirals used in pregnancy were stratified in two types of regimens, PI-based and NNRTI-based, showing 224 newborn cases exposed to PIs and 18 to NNRTIs. Data details about therapy were missing in eight cases, and in seven cases, there was no gestational date recorded. A total of 105 newborns were not exposed to antiretrovirals in utero (Table 2).

The distribution of third antiretroviral drugs among the studied groups differed significantly, mainly due to missing data on gestational age group, so the results were not interpretable under the chosen model (*X*^2^ (6, *N* = 352) = 14.23, *p* = 0.02).

Further analysis of different PI molecules’ distribution among the studied groups showed no statistically significant difference (*X*^2^ (6, 224) = 10.77, *p* = 0.09) (Table 2). In cases treated with NNRTI-based regimens (*X*^2^ (2, 15) = 1.2, *p* = 0.54), no relationship between any molecule and preterm birth was found. Regarding the backbone combinations used in cART during pregnancy, no statistically significant difference between preterm and full-term delivery was observed (*X*^2^ (10, 352) = 17.52, *p* = 0.06).

#### 3.1.4. Low Birth Weight in Full-Term Newborns Assessment

The body weight at birth was known in 263 out of 269 full-term newborns, 33 newborns (12.26%) were small for their gestational age, and in 6 cases (2.5%), data about birth weight was missing. The characteristics of patients are detailed in Table 3.

Low birth weight distribution in full-term babies was similar between genders, *X*^2^ (2, 269) = 2.78, *p* = 0.24).

Mean maternal age in the overall population of studied women (269) was 23 years (SD = 4.3916, 95%CI = 22.4728–23.5272). In the group of women who gave birth to low-birth-weight full-term babies, it was also 23 years (SD = 4.75737, 95%CI = 21.3131–24.6869), as well as in the groups with normal-birth-weight full-term babies (SD = 4.3713, 95%CI = 22.4321–23.5679). Overlapping 95%CIs in mean maternal age showed no significant differences among means in the studied groups, confirmed by the *t* test for variance results, *p* = 0.67.

In a univariate analysis, low birth weight in full-term infants was associated with HIV-positive final status (*X*^2^ (4, 269) = 11.23, *p* = 0.02) and lack of maternal cART during pregnancy (*X*^2^ (4, 269) = 9.84, *p* = 0.04) (Table 3).

The uneven distribution of maternal chronic HBV infection rate was due to the high rate in the group with missing data on gestational age, precluding an interpretable conclusion (*X*^2^ (2, 269) = 7.89, *p* = 0.01). The other studied maternal comorbidities, chronic HCV infection (*X*^2^ (2, 269) = 1.05, *p* = 0.59), illegal drug use (*X*^2^ (2, 269) = 0.77, *p* = 0.67), and syphilis diagnosis during pregnancy (*X*^2^ (2, 269) = 2.06, *p* = 0.35), were not associated with low or normal birth weight in the studied patients (Table 3).

The type of delivery, namely vaginal delivery and planned or necessary cesarean section, revealed no significant association with low or normal birth weight in full-term babies (*X*^2^ (6, 269) = 9.51, *p* = 0.14).

The other analyzed variables revealed no statistically significant differences, as presented in Table 3. Maternal HIV diagnosis timing was stratified as preconception, during pregnancy, and during or after birth, with a computed Pearson *X*^2^ of 1.79, (4, 269), *p* = 0.77.

Maternal antiretroviral initiation timing, stratified as preconception, during pregnancy, and during or after birth, had a computed Pearson *X*^2^ of (6, 269) = 7.55, *p* = 0.47. Statistical analysis for maternal HIV RNA levels and CD4 count levels found no association with low birth weight in full-term delivery (*X*^2^ (8, 269) = 6.1, *p* = 0.63; *X*^2^ (6, 269) = 4.72, *p* = 0.57). Data regarding maternal antiretroviral treatment before conception in terms of duration and number of regimens showed non-statistically significant differences among the studied groups (low- and normal-birth-weight babies) (*X*^2^ (10, 269) = 7.63, *p* = 0.66 and *X*^2^ (10, 269) = 17.12, *p* = 0.07, respectively).

The mean number of antiretroviral regimens before conception differed significantly among the studied groups (test for variance chi square = 21.16, *p* = 0.00002). This indicated that a higher number of regimens before conception was associated with a higher likelihood of normal birth weight in full-term gestation in the studied patients.

Exposure to antiretrovirals during the first trimester of pregnancy was not statistically significantly associated with low birth weight (*X*^2^ (6, 269) = 5.56, *p* = 0.27).

PIs and NNRTIs had a similar distribution among babies with low and normal birth weight, and both classes had the same likelihood of being associated with low or normal birth weight (*X*^2^ (6, 269) = 5.442, *p* = 0.48). A similar result was yielded in the statistical analysis regarding specific PI molecules (boosted lopinavir, boosted saquinavir, and nelfinavir) (*X*^2^ (6, 168) = 8.56, *p* = 0.19), NNRTI molecules (nevirapine, efavirenz, and etravirine) (*X*^2^ (2, 15) = 0.2679, *p* = 0.87), and backbone combinations (*X*^2^ (10, 269) = 7.17, *p* = 0.70) (Table 4).

## 4. Discussion

The rate of HIV vertical transmission noted in this study, 13.9%, is higher than the reported mother-to-child transmission for the same period at the national level, around 9%, and discordant with data reported from other Romanian centers, with a rate of vertical transmission ranging from 5.4% to 6.97% [18,25]. These discrepancies could be explained by the study design and selection criteria, the addressability to study sites, and regional differences in patients’ access to the National Program for mother-to-child HIV transmission prevention [20].

The rate of preterm birth in our study population was 21.5%, 2.2 times greater than European and Romanian average data (around 10%) for the general population in the same time period [26]. This suggests the need for more efficient strategies to improve the medical and nursing care provided to HIV-infected pregnant women to be able to assist this type of patient. Similarly high rates of premature birth have been reported in other HIV cohorts, as follows: in an Italian Cohort, the rate was 22.6% versus 11.9% in uninfected women, data published in 2014, and in a US-based cohort of 1869 newborns, the rate of premature delivery was 18.6%, where it was associated with use of protease inhibitor-based cART [12,14].

In this study, in the univariate analysis, the preterm birth rate was associated with HIV transmission in studied newborns and with high levels of maternal HIV RNA in the final trimester, suggesting that a lack of viral replication control was a predictive factor for preterm delivery in our patients. These results cannot be generalized to Romanian HIV-exposed newborns due to the low statistical power of our study.

Nevertheless, the role of HIV replication and advanced HIV diseases in birth outcomes, like prematurity and low birth weight, has been noted in previous studies. Schulte and collaborators assessed the trends of prematurity and low birth weight in 11,231 infants during five years, 1998–2004, showing a decrease in low birth weight rate in infants from 35% to 21% and prematurity rate from 35% to 22%, while the use of antiretrovirals in pregnant women increased from 2% to 97%. They concluded that maternal use of illegal drugs, unknown maternal HIV status before delivery, symptomatic maternal infection before delivery, and HIV infection in infants were associated with low birth weight and prematurity. These results are similar with the data observed in our study [27].

In the above-mentioned results published by Schulte and colleagues, prematurity was associated with a lack of cART during pregnancy and the use of protease inhibitor regimens, while our results found no relationship with any antiretroviral regimen. This discordance in the results can be explained by the study design, retrospective versus longitudinal, selection criteria, with patients reporting to two major centers, comparative to a pediatric consortium covering multiple sites, the number of studied patients, 358 versus 11,321, and a study period of six years (2006–2012) versus 14 years (1989 till 2004) [27].

Meanwhile, a study published in 2014 by Slyker and colleagues found similar results with our study, showing a correlation between detectable maternal HIV RNA levels and prematurity risk, with an odds ratio (OR) of 1.8. At the same time, this study showed an increased prematurity rate of 3.3 times associated with less than 15% CD4, while in our study, the data showed no relationship with CD4 levels. These differences can be explained by the differences in inclusion criteria, namely that HIV-infected infants were included in our study, but excluded in Slyker’s study [28].

To the best of our knowledge, this study is the first to assess the impact on birth outcomes in Romanian women exposed to antiretrovirals since childhood, including during puberty.

Nevertheless, in our study, duration of therapy prior to pregnancy and class of ARV used during pregnancy, protease inhibitors or NNRTI, were not associated with a higher rate of preterm delivery or low birth weight in newborns exposed to maternal cART. Similar results from a study with a Swiss cohort comprising 1180 pregnancies, with a rate of preterm birth of 15.8% before 1994 and 28% after 1998, also showed no relationship between the duration before delivery and rate of premature birth, but the mean age of studied women was 29 years, comparatively higher than our study population [29].

In our study, the higher number of antiretroviral regimens was significantly associated with normal birth weight. These results could have been influenced by confounders like better monitoring and control of HIV infection in women with multiple regimens, or survivor bias, factors not assessed in our study. Divergent from our results, a study published in 2012, including 183 women with a mean age of 28 years, found that the prevalence of babies small for their gestational age was 31.2% at the 10th percentile and 12.6% at the 3rd percentile, and there was no association with treatment initiation before pregnancy [30].

In contrast to our results, a more recent study carried out in France found a preterm birth rate of around 14% in INSTI-treated pregnant women compared to 21% in our patients. This difference could be explained by the dissimilar study design, prospective versus retrospective, national versus local [31]. At the same time, comparing the French study’s results to the median European preterm birth rate of around 5% (moderate and late preterm birth in 100 live births), ranging from 4% to 7.65%, the rate was still two times higher in the French HIV cohort, even with newer antiretroviral drugs, INSTIs [32].

Similar data to that of French cohort was published on AIDS in 2022 from a study conducted in the South African Republic including 991 pregnant women, showing a rate of 14% preterm birth in HIV-exposed but noninfected newborns compared to 9.8% in HIV-negative pregnant women [33]. At the same time, in the South African study, the low birth weight rate was higher in HIV-exposed compared to unexposed infants, 14.3% versus 11.4%, with the difference being more important for very low birth weight, 3.7% versus 1.57%, suggesting that HIV played an important role in birth outcomes in the studied African population.

Moreover, a meta-analysis published in 2023 showed uncertainties in connection with preterm delivery and in relation to antiretrovirals, despite the fact that relation to protease inhibitor exposure, mainly lopinavir/ritonavir during pregnancy, has been proven by many studies. Newer protease inhibitors, like darunavir–ritonavir, have limited data regarding safety during pregnancy. The same issues were found by the previously mentioned metanalysis for timing of cART initiation and relation with premature birth and low birth weight [34]. The authors conclude that there was no clear association between different antiretroviral regimens, including the newer one based on integrase inhibitors, and prematurity and low birth weight, so clinicians should continue to monitor and report any adverse events in HIV pregnant women.

In our study, the rate of low birth weight in HIV-exposed children was 12.26% in full-term newborns. In the univariate analysis, the low birth weight rate in the studied patients was associated with HIV-positive final status and a lack of maternal antiretroviral treatment during pregnancy, suggesting the same important impact of HIV on fetal growth in our studied population. Our results, despite being similar with those mentioned before, published by Shulte and collaborators, could not be extrapolated to other cohorts due to the limits of our study, namely the retrospective design, the inclusion bias, and multifactorial nature of birth outcomes, not fully assessed in this paper [27].

At the same time, in our study, low birth weight in full-term newborns was not associated with HCV coinfection and illegal drug use in pregnant women. Our results differed from a meta-analysis and systematic review published in 2022 in Lancet by Cowdell and collaborators, showing that PI-based regimens were associated with a high risk of being small for gestational age (SGA). These different results could be explained by the retrospective design of our study, inclusion criteria for the patients, and possible regional particularities, while the meta-analysis included numerous studies from different geographical regions [35].

The underlying mechanism implicated in explaining the excess of premature birth in HIV-treated pregnant women could be the impact of antiretroviral drugs on mitochondrial DNA and immunomodulation. The results of a study on mitochondrial DNA (mitDNA) in exposed infants showed surprising data. Levels of mitDNA from peripheral monocytes (PBMCs) were higher in ARV-exposed versus ARV-unexposed children [36]. Immunomodulation-associated antiretroviral treatment is characterized by increased production of IL2, which is favorable for controlling HIV disease, but unfavorable for pregnancy maintenance [37].

The factors implicated in preterm birth in HIV-treated women are probably more complex, and further randomized and large studies are needed for a better description of these mechanisms.

At the same time, birth weight is driven by many complex elements, including genetic and environmental factors. The multifaceted nature of birth weight was described by Libretti and collaborators in a recent review of the literature, including nineteen publications showing the important role of maternal and paternal characteristics. Maternal characteristics found to be involved in determining fetal growth were age, BMI, weight gain during pregnancy, number of prior pregnancies, and socioeconomic status. But at the same time, paternal variables were found to play a significant role in fetal growth, including paternal age at conception and paternal height. Paternal BMI and weight were not clearly associated with specific aspects of fetal growth. These data show the multicausal nature of fetal growth and birth weight. In our study, we did not assess for paternal characteristics, which would be a very interesting theme for future studies [38].

Study limitations were mostly due to the retrospective observational design; analysis has been based on pre-collected information, introducing important constraints regarding data quality, selection bias, and relatively high amount of missing data. The first major limitation was the selection bias, namely including only individuals monitored in two major medical centers. We did not have a control group from the general population and we used published statistical data for the general population, so we could not control the confounding variables affecting our study population and potentially the outcomes. The second major limitation was the incomplete information in digital medical records, since we used real-world records where the main focus was on medical care. A relatively high rate of missing data of 11% for HIV final status could have had an important impact on the HIV transmission rate, noted as 13.9% from available information. Another limitation of the study was the inclusion of all live births of one woman if they were in the analyzed time frame, potentially introducing a cluster effect.

## 5. Conclusions

This study demonstrated that in pregnant women that have lived with HIV from their early childhood and are multi-drug experienced, prematurity was mainly associated with the level of viral replication during the last trimester of pregnancy and HIV vertical transmission. Low birth weight in newborns was associated with a lack of antiretroviral treatment during pregnancy and HIV transmission. The study results suggest that HIV itself contributes more substantially to adverse birth outcomes compared to antiretroviral therapy, irrespective of the treatment duration and number of regimens before conception, nor exposure throughout puberty in future mothers.

Antiretroviral therapy represents the cornerstone of prevention for HIV vertical transmission by effectively suppressing viral replication; long-term exposure to ARV does not influence birth outcomes.

Monitoring pregnancy and good access to HIV prevention programs are the main issues that have to be addressed in Romania. The rate of very low birth weight in the studied patients is a concern, but the solution is to improve the quality of medical care for HIV-infected women at fertile age.

Several limitations of this study have to be taken into account, including the design as a retrospective observational study, the relatively low number of patients studied, and the absence of information concerning other maternal conditions that can impact on birth outcome like tobacco and alcohol use.

Nevertheless, these results provide useful information from real-world settings in an area of medical care still under debate, making it difficult to develop appropriate guidelines for Romania and other countries with similar epidemics.

## Figures and Tables

**Table 1:** Correlations of birth outcome (preterm and full-term delivery) in HIV vertically exposed children.

Children’s Characteristics	Preterm Birth (N = 76, 21.59%)	Full-Term Birth (N = 269, 76.42%)	Missing Data (N = 7, 1.98%)	Statistical Significance
Gender—Male	40 (52.63%)	149 (55.39%)	2 (28.57%)	0.35 ^a^
HIV final status				0.001 ^a^
Negative	49 (64.47%)	210 (78.06%)	3 (42.85%)	
Positive	14 (18.42%)	29 (10.78%)	4 (57.14%)	
Lost to follow-up	13 (17.10%)	30 (11.15%)	0	
Maternal Characteristics				
Comorbidities				
Chronic HBV coinfection	8 (10.525)	23 (8.55%)	1 (14.28%)	0.77 ^a^
Chronic HVC confection	4 (5.26%)	9 (3.34%)	1 (14.28%)	0.27 ^a^
History of illegal drug use	7 (9.21%)	10 (3.71%)	0	0.11 ^a^
Syphilis	2 (2.63%)	13 (4.83%)	0	0.60 ^a^
Obstetric complications	2 (2.63%)	0	0	0.02 ^a^
Type of delivery				
Vaginal	34 (44.73%)	76 (28.25%)	1 (14.28)	<0.0001 ^a^
Planned C section	22 (28.94%)	171 (63.56%)	0	
Necessary C section	17 (22.36%)	2 (0.74%)	0	
Missing data	3 (3.94%)	20 (7.43%)	6 (85.71)	
HIV diagnosis				0.26 ^a^
Before conception	50 (65.78%)	169 (62.82%)	2 (28.57%)	
During pregnancy	11 (14.47%)	54 (20.07%)	1 (14.28%)	
At or after birth	15 (19.73%)	46 (17.1%)	4 (57.14%	
CD4 count in third trimester (cells/mm^3^)				0.08 ^a^
<200	12 (15.78%)	34 (12.63%)	1 (14.28%)	
200–500	25 (32.89%)	98 (36.43%)	1 (14.28%)	
>500	22 (28.94%)	96 (35.68%)	1 (14.28%)	
Missing data	17 (22.36%)	41 (15.24%)	4 (57.14%)	
HIV RNA level in third trimester or at birth (copies/mL)				0.004 ^a^
<50	7 (9.21%)	74 (27.5%)	0	
50–200	2 (2.63%)	9 (3.34%)	0	
201–1000	10 (13.15%)	17 (6.31%)	2 (28.57%)	
>1000	34 (44.73%)	94 (34.94%)	1 (14.28%)	
Missing data	23 (30.26%)	75 (27.88%)	4 (57.14%)	
Timing of cART initiation				0.007 ^a^
Preconception	42 (55.26%)	142 (52.78%)	1 (14.28%)	
During pregnancy	14 (18.42%)	52 (19.33%)	1 (14.28%)	
At or after birth	11 (14.47%)	63 (23.42%)	5 (71.42%)	
Missing data	9 (11.84%)	12 (4.46%)	0	
Duration of cART before conception				0.44 ^a^
>120 months	8 (19.04%)	24 (16.9%)	1 (100%)	
60–120 months	15 (35.71%)	61 (42.95%)	0	
12–59 months	11 (26.19%)	26 (18.3%)	0	
<12 months	3 (7.14%)	5 (3.52%)	0	
Missing data	5 (11.9%)	26 (18.3%)	0	
Total	42 (100%)	142 (100%)	1 (100%)	
cART in pregnancy				0.04 ^a^
Yes	50 (65.78%)	187 (69.51 %)	2 (28.57%)	
No	22 (28.94%)	78 (28.99%)	5 (71.42%)	
Missing data	4 (5.26 %)	4 (1.48%)	0	
Timing cART exposure in pregnancy				0.30 ^a^
Before 12 weeks	42 (55.26%)	142 (52.78%)	1 (14.28%)	
After 12 weeks	13 (17.1%)	43 (15.98%)	1 (14.28%)	
Missing data	2 (2.63%)	5 (1.85%)	0	
None	19 (25%)	79 (29.36%)	5 (71.42%)	
Number of combinations before conception				0.50 ^a^
0	29 (38.15%)	99 (36.80%)	6 (85.71%)	
1	11 (14.47%)	54 (20.07%)	0	
2	15 (19.73%)	43 (15.98%)	0	
3	10 (13.15%)	34 (12.63%)	1 (14.28%)	
More than 3	8 (10.52%)	28 (10.40%)	0	
Missing data	3 (3.94%)	11 (4.08%)	0	
Mean	1.418919	1.501845	0	0.16 ^b^
SD	±1.412598	±1.646263	±1.1238	

Notes—^a^ Pearson chi square test, ^b^ ANOVA *t* test.

**Table 2:** Correlations with antiretroviral drugs and prematurity in HIV vertically exposed children.

Antiretroviral Drug Type Used in Pregnancy	Preterm Birth (N = 76, 21.59%)	Full-Term Birth (N = 269, 76.42%)	Missing Data (N = 7, 1.98%)	Statistical Significance
Antiretroviral classes for the 3rd drug				0.02 ^a^
PI	46 (60.52%)	173 (64.31%)	2 (28.57%)	
INNRT	3 (3.94%)	15 (5.57%)	0	
None	22 (28.94%)	78 (28.99%)	5 (71.42%)	
Missing data	5 (6.57%)	3 (1.11%)	0	
Total	76 (100%)	269 (100%)	7 (100%)	
Type of PI for the 3rd drug				0.09 ^a^
Lopinavir/ritonavir	36 (73.46%)	128 (91.32%)	1 (50%)	
Saquinavir/ritonavir	11 (22.44%)	27 (15.6%)	0	
Nelfinavir	1 (2.04%)	10 (5.78%)	1 (50%)	
Other	1 (2.04%)	8 (4.62%)	0	
Total	49 (100%)	173 (100%)	2 (100%)	
Type of NNRTI				0.54 ^a^
Efavirenz	1 (33.33%)	5 (33.33%)	0	
Nevirapine	2 (66.66%)	6 (40%)	0	
Etravirine	0	4 (26.66%)	0	
Total	3 (100%)	15 (100%)	0	
Type of Backbone				0.06 ^a^
AZT + 3TC	41 (53.94%)	143 (53.15%)	2 (28.57%)	
ABC + 3TC	3 (3.94%)	22 (8.17%)	0	
TDF + 3TC	2 (2.63%)	3 (1.11%)	0	
Other	3 (3.94%)	20 (7.43%)	0	
No	22 (28.94%)	78 (28.99%)	5 (71.42%)	
Missing	5 (6.57%)	3 (1.11%)	0	
Total	76 (100%)	269 (100%)	7 (100%)	

Notes—^a^ Pearson chi square test.

**Table 3:** Patient characteristics in full-term newborns by birth weight.

Variables	Low Birth Weight (N = 33, 12.26%)	Normal Birth Weight (N = 230, 85.5%)	Mising Birth Weight Data (N = 6, 2.5%)	Statistical Significance
Children Characteristics				
Gender				
Male	14 (42.42%)	131 (36.95%)	4 (66.66%)	0.24
HIV final status				0.02
Negative	28 (84.84%)	179 (77.82%)	3 (50%)	
Positive	3 (9.09%)	23 (10%)	3 (50%)	
Indeterminate	2 (6.06%)	28 (12.17%)	0	
Total	33 (100%)	230 (100%)	6 (100%)	
Maternal Characteristics				
Comorbidities				
Chronic HBV coinfection	0	21 (9.1%)	2 (33.33%)	0.01
Chronic HVC confection	2 (6.06%)	7 (3.04%)	0	0.59
History of illegal drug use	2 (6.06%)	8 (3.47%)	0	0.67
Syphilis	1 (3.03)	11 (4.78%)	1 (16.66%)	0.35
Type of delivery				0.14
Vaginal	13 (39.39%)	61 (26.52%)	2 (33.33%)	
Planned C section	19 (57.57%)	150 (63.55%)	2 (33.33%)	
Necessary C section	0	2 (0.86%)	0	
Missing data	1 (3.03%)	17 (7.39%)	2 (33.33%)	
Moment of HIV diagnosis				
Preconception	19 (57.57%)	147 (63.91%)	3 (50%)	0.77
During pregnancy	7 (21.21%)	46 (20%)	1 (16.66%)	
At or after birth	7 (21.21%)	37 (16.08%)	2 (33.33%)	
Total	33 (100%)	230 (100%)	6 (100%)	
CD4 levels in third trimester (cells/mm^3^)				0.57
<200	7 (21.21%)	27 (11.73%)	0	
200–500	10 (30.3%)	86 (37.39%)	2 (33.33%)	
>500 cells/mm^3^	12 (36.36%)	82 (35.65%)	2 (33.33%)	
Missing data	4 (12.12%)	35 (15.21%)	2 (33.33%)	
Total	33 (100%)	230 (100%)	6 (100%)	
HIV RNA levels in third trimester (copies/mL)				
<50	8 (24.24%)	65 (28.26%)	1 (16.66%)	0.63
50–200	3 (09.09%)	6 (2.60%)	0 (%)	
200–1000	3 (09.09%)	13 (5.65%)	1 (16.66%)	
>1000	11 (33.33%)	81 (35.61%)	2 (33.33%)	
Missing data	8 (24.24%)	65 (28.26%)	2 (33.33%)	
Total	33 (100%)	230 (100%)	6 (100%)	
Moment of cART initiation				0.27
Preconception	17 (51.51%)	124 (53.91%)	1 (16.66%)	
During pregnancy	5 (15.15%)	46 (20%)	1 (16.66%)	
At or after birth	9 (27.27%)	51 (22.17%)	4 (66.66%)	
Missing data	2 (6.06%)	9 (3.91%)	0	
Total	33 (100%)	230 (100%)	6 (100%)	
Duration of antiretroviral therapy before pregnancy				
>120 months	1 (3.03%)	23 (10%)	0 (%)	0.66
60–120 months	6 (18.18%)	54 (23.47%)	1 (16.66%)	
12–59 month	4 (12.12%)	22 (9.56%)	0 (%)	
<12 month	0	5 (2.17%)	0 (%)	
None	17 (51.51%)	101 (43.91%)	5 (83.33%)	
Missing	5 (15.15%)	25 (10.86%)	0 (%)	
Total	33 (100%)	230 (100%)	6 (100%)	
Antiretroviral treatment during pregnancy				0.043
Yes	9 (27.27%)	64 (27.82%)	4 (66.66%)	
No	21 (63.63%)	106 (46.09%)	1 (16.66%)	
Missing data	3 (0.91%)	60 (26.09%)	1 (16.66%)	
Total	33 (100%)	230 (100%)	6 (100%)	
Exposure to antiretroviral drugs during pregnancy				0.47
In the first 12 weeks	16 (48.48%)	125 (54.34%)	1 (16.66%)	
After 12 weeks	6 (18.18%)	36 (15.65%)	1 (16.66%)	
Missing data	0	4 (1.73%)	0	
None	11 (33.33%)	65 (28.26%)	4 (66.66%)	
Total	33 (100%)	230 (100%)	6 (100%)	
Number of combinations before pregnancy				
0	9 (27.27%)	85 (39.95%)	5 (%)	0.071
1	13 (39.39%)	41 (17.82%)	0	
2	6 (18.18%)	36 (15.65%)	1 (16.66%)	
3	2 (6.06%)	32 (13.91%)	0	
More than 3	2 (6.06%)	32 (13.91%)	0	
Missing data	1 (3.03%)	4 (1.73%)	0	
Total	33 (100%)	230 (100%)	6 (100%)	
Mean	1	2	0	0.00001
SD	±1	±2	±1	
95%CI	0.64–1.35	1.74–2.25	−1.04–1.04	

**Table 4:** Distribution of antiretroviral therapy used in full-term babies by birth weight.

Type of Antiretroviral Drug Used in Pregnancy	Low Birth Weight (N = 33, 12.26%)	Normal Birth Weight (N = 230, 85.5%)	Mising Birth Weight Data (N = 6, 2.5%)	Statistical Significance
Antiretroviral classes of 3rd drug				
PI	21 (63.63%)	145 (63.04%)	2 (33.33%)	0.48
NNRTI	3 (9.09%)	12 (5.21%)	0 (%)	
Other	1 (3.03%)	8 (3.47%)	0 (%)	
None	8 (24.24%)	65 (28.26%)	4 (66.66%)	
Total	33 (100%)	230 (100%)	6 (100%)	
Type of PI				
Lopinavir/ritonavir	15 (65.21%)	107 (73.79%)	1 (16.66%)	0.19
Saquinavir/ritonavir	2 (8.69%)	8 (5.51%)	1 (16.66%)	
Nelfinavir	2 (8.69%)	24 (16.55%)	0 (%)	
Other PI	2 (8.69%)	6 (4.13%)	0 (%)	
Total	23 (100%)	145 (100%)	2 (100%)	
Type of NNRTI				0.87
Efavirenz	1 (33.33%)	3 (25%)	0	
Nevirapine	1 (33%)	6 (50%)	0	
Etravirine	1 (%)	3 (25%)	0	
Total	3 (100%)	12 (100%)	0	
Type of Backbone				0.70
AZT + 3TC	18 (54.54%)	123 (53.47%)	2 (33.33%)	
ABC + 3TC	4 (12.12%)	18 (7.82%)	0 (%)	
TDF + 3TC	1 (3.03%)	2 (0.86%)	0 (%)	
Other	2 (6.06%)	18 (7.82%)	0 (%)	
No	8 (24.24%)	66 (28.69%)	4 (66.66%)	
Missing	0	3 (1.30%)	0	
Total	33 (100%)	230 (100%)	6 (100%)	

## Data Availability

The datasets supporting our research published here are available on request from the corresponding authors.
